# Azerbaijani adaptation of the WHO-5 wellbeing index: investigating its relationship with psychological distress, resilience, and life satisfaction

**DOI:** 10.1186/s40359-024-01593-0

**Published:** 2024-02-27

**Authors:** Bakhtiyar Aliyev, Elnur Rustamov, Seydi Ahmet Satici, Ulkar Zalova Nuriyeva

**Affiliations:** 1 Psychology Scientific Research Institute, Ziya Bünyadov, 38, Nərimanov, AZ1075, Baku, Azerbaijan; 2 Faculty of Education, Department of Psychological Counselling, Yildiz Technical University, Istanbul, Türkiye

**Keywords:** Wellbeing, Distress, Resilience, Scale adaptation

## Abstract

**Background:**

The WHO-5 Wellbeing Index is a widely used tool for assessing psychological well-being. Despite its global application, its adaptation and validation for the Azerbaijani population had not been previously explored. This study aims to fill this gap by adapting the WHO-5 Wellbeing Index for Azerbaijani adults and examining its relationship with psychological distress, resilience, and life satisfaction.

**Methods:**

A sample of 875 Azerbaijani adults aged 18 to 89 (mean age = 29.13, SD = 10.98) participated in this study. The adaptation process included confirmatory factor analysis to test the original 5-item structure of the index in the Azerbaijani context. Additionally, item response theory analysis was employed to evaluate the discriminative values of the items. Reliability was assessed through various methods, including Cronbach’s alpha, McDonald’s omega, and Guttmann’s lambda.

**Results:**

Confirmatory factor analysis supported the original 5-item structure of the WHO-5 Wellbeing Index for the Azerbaijani sample, demonstrating alignment with the index’s original version. All items showed acceptable discriminative values in item response theory analysis. The index also exhibited sufficient reliability, as evidenced by Cronbach’s alpha, McDonald’s omega, and Guttmann’s lambda. Correlation and network analyses indicated significant associations of the WHO-5 Wellbeing Index with psychological distress, resilience, and life satisfaction. Specifically, the index was negatively correlated with depression, anxiety, and stress, and positively correlated with resilience and life satisfaction.

**Conclusion:**

The findings suggest that the WHO-5 Wellbeing Index is a valid and reliable tool for assessing psychological well-being in the Azerbaijani population. Its significant associations with psychological distress, resilience, and life satisfaction further affirm its utility in this cultural context.

## Background

The level of well-being, synonymous with mental health, is considered a state characterized by positive feelings, high quality of life, and life satisfaction [1, 2]. In other words, well-being is considered as a state in which people experience positive emotions, feel satisfied, are able to work and study productively, realize their potential, and also have some control over their life and the events that occur therein [3]. Research indicates that individuals who are satisfied with their relationships, successful in their careers and education, and enjoy their personal lives exhibit higher levels of well-being [4, 5]. Diener et al. conceptualize well-being as a cognitive and affective evaluation of oneself and one’s life, which includes cognitive assessments and emotional responses to life’s events [6, 7]. Over the years, researchers have taken different approaches to study and measure the concept of well-being. Huppert and So, in their systematic approach to measuring individuals’ well-being, suggest that high well-being and mental health should be considered as the antithesis of pathologies or mental illnesses [8]. This implies that the presence of a state of well-being could reflect a decrease in the symptoms of widespread mental disorders within society [9].

In any psychological context, the presence of well-being, whether at high or low levels, significantly influences an individual’s mental health, as well as their orientation and attitudes toward life, events, and more [10]. Individuals who enjoy a high level of well-being stand out in society through their social behavior and the establishment of positive, sincere relationships. They exhibit higher levels of self-confidence, increased creativity, and more effective functioning in their work and learning activities. Additionally, these individuals put more effort into achieving their goals and spend their days more productively [8, 11]. Research in this area suggests that experiencing a high level of well-being during childhood is predictive of maintaining a high level of well-being in the future [9, 12].

It is undeniable that individuals with a low level of well-being are more susceptible to depression and stress, conditions often associated with the occurrence of suicide attempts or self-injury [13–15]. Several researchers [16] have posited that a predisposition towards pessimism over optimism adversely affects life satisfaction and happiness, thereby elevating levels of depression and stress. Such a state further escalates the risk of experiencing both physical and mental health issues, complicating the swift recovery from and resolution of these conditions [17]. Factors such as exposure to violence, poor living conditions, the inability to recognize value in oneself and loved ones, and persistent failure are among the most significant detractors from well-being.

Another concept related to well-being is psychological resilience. Resilience is defined as the capacity for rapid recovery and the re-establishment of normal functioning after being subjected to stress-inducing life events that lead to a breakdown in functionality [18]. Conceptually, it embodies the strength to remain steadfast in the face of adversity, skillfully managing challenging situations without yielding to despair [19]. This concept is characterized by a dynamic adaptation mechanism, marked by positive adjustment patterns in response to adverse conditions, which evolve over time [20, 21]. Such a construct is crucial for therapeutic interventions aimed at addressing maladaptive reactions to anxiety and depression, highlighting its importance in promoting psychological resilience [22]. Additionally, research by Ong et al. [23] has shed light on the ability of highly resilient individuals to effectively recover from daily stress, suggesting resilience as a key predictor of enhanced well-being. Likewise, individuals with a higher level of resilience have shown significantly more positive cognitive patterns and reported higher levels of well-being [24]. Therefore, resilience not only aids in coping and adapting in adverse situations but also plays a significant role in improving the well-being of individuals.

To study and measure the well-being of individuals across different cultures, standard measurement tools that are equivalent in terms of language and concept are essential. Although there are several tools available to assess people’s well-being, the WHO-5 Well-Being Index, adapted into more than 30 languages, has emerged as one of the most convenient and widely utilized scales [25, 26]. Analyzing the structure of this scale in the Azerbaijani language, and verifying its psychometric properties such as validity and reliability, is crucial. Such analysis is necessary to understand the orientation of adults in Azerbaijan towards well-being and to accurately determine their well-being levels. The well-being index scale is a self-rated tool that captures positive feelings and measures subjective well-being and its dimensions based on individuals’ states over the past 14 days [27]. Originally developed for use in healthcare settings to assess patients’ depressive symptoms and suicidal tendencies, the scale’s psychometric properties were first examined in clinical contexts, including patient populations in clinics and hospitals, where it gained significant importance [28, 29]. The WHO-5 well-being scale, a concise instrument consisting of 5 items, evolved from the WHO-10. The WHO-10 itself derives from a 28-item version of the well-being index scale that was used in a study by the World Health Organization across eight different European countries [30].

WHO-5 Well-Being Index consisting of 5 items has been adapted into more than 30 languages, including Chinese [31], Persian [32], Sinhala [30], Norwegian [33], and Thai [34]. In previous studies examining the structure of the WHO-5 Well-being Index, it was similarly confirmed among Chinese university students that the 5-item scale was unidimensional, with reliability coefficients reported as 0.85 and 0.81 across two different datasets [31]. In a different group, consisting of infertile patients, the unidimensional structure of the scale was also confirmed in the examined Persian version, and the Cronbach’s alpha coefficient was determined to be 0.86 [32]. When looking at the results for the Norwegian version, which was examined with caregivers, it was reported that the confirmatory factor analysis results showed a good fit, and the Cronbach’s alpha coefficient was similarly noted to be 0.86 [33]. In addition, the psychometric properties of the scale were evaluated during a cross-sectional study that spanned three countries—Spain, Chile, and Norway—to assess individuals’ well-being levels during the COVID-19 pandemic [35]. Studies conducted in various countries have revealed that cultural differences significantly influence the assessment of well-being levels across different cultures. The analysis of the WHO-5 well-being scale’s structure in numerous countries and the verification of its psychometric properties highlight its vital role in evaluating individuals’ well-being. The absence of a tool for measuring well-being in the Azerbaijani language represents a considerable gap in research. Therefore, adapting the WHO-5 Well-Being Index to the Azerbaijani context, along with a comprehensive examination of its structure through various statistical analyses and validation of its psychometric properties, such as validity and reliability, is crucial. This effort goes beyond merely enriching the scientific literature; it establishes a foundation for preventive strategies by facilitating the assessment of adults’ well-being levels. Accordingly, this research not only aimed at the scale’s adaptation but also explored the relationship between well-being and factors such as depression, anxiety, stress, resilience, and life satisfaction among Azerbaijani individuals.

## Method

### Participants

The research involved 875 participants, ranging in age from 18 to 89 years (with a mean age of 29.13 years and a standard deviation of 10.98). Of the participants, 754 were female (86.2%) and 121 were male (13.8%). Out of the participants, 374 were in a marital union (42.7%), while 501 were single (57.3%). A majority of the participants had attained higher education levels (*n* = 739, 85%). In the study, the employment status of participants was categorized as follows: 476 individuals (54.4%) were employed, while 399 individuals (45.6%) were not employed, all of whom were students. A substantial percentage, specifically 77.4%, perceived their socioeconomic status as medium, with 16.8% considering it as low and 5.8% as high. Detailed information regarding the participants is presented in Table 1.

**Table 1:** Participants’ characteristics

Variable	Frequency	%
*Gender*		
Female	754	86.2
Male	121	13.8
*Marital Status*		
Married	374	42.7
Single	501	57.3
*Educational Status*		
High school	56	6.4
Vocational or technical secondary education	80	9.1
Higher education	739	84.5
*Employment Status*		
Employed	476	54.4
Not employed (students)	399	45.6
*Perceived Socio*-*Economic Status*		
Poor	147	16.8
Moderate	677	77.4
Good	51	5.8

### Ethics

The study adhered rigorously to the ethical principles outlined in the Helsinki Declaration. Before initiating the research, ethical approval was obtained from the Psychology Scientific Research Institute Ethics Committee (ID: T-474), Baku, Azerbaijan. Informed consent was obtained from all the individual participants that were included in the study.

### Measures

**The WHO-5 Well-being Index** was developed by WHO [36] to evaluate subjective well-being. This scale is a non-symptomatic and positively worded self-report tool, which consists of five statements (“I have felt cheerful and in good spirits”, “My daily life has been filled with things that interest me”). The degree to which these feelings were present over a 14-day period was scored on a 6 -point Likert-type scale ranging from 0 = “at no time” to 5 = “all of the time”. According to the results, an individual score ranging from 0 to 25, with lower scores indicating lower levels of well-being. As scales measuring health-related quality of life are conventionally converted to a percentage point, the summed score was multiplied by 4 to convert from 0 to 100 points.

**Satisfaction with Life Scale (SWLS)** is a brief assessment scale designed by Diener, Emmons, Griffin and Larsen [37, 38]. The Azerbaijani version of the SWLS was conducted by Osmani et al. [39]. The SWLS contains 5 items (e.g., “If I could live my life over, I would change almost nothing”) to evaluate one’s satisfaction with life with a 7-point Likert scale (from 1 “strongly disagree” to 7 “strongly agree”). The higher scores indicated a higher level of satisfaction with life. The reliability analysis showed that Cronbach’s alpha index of internal consistency was 0.74 in this sample.

#### Depression, anxiety and stress scale − 21 items (DASS-21)

The DASS-21 [40, 41] created symptoms of depression, anxiety, and stress. The Azerbaijani version of the DASS-21 was conducted by Rustamov et al. [42]. The scale composed of 21 items and three subscales, each with seven items (e.g., “I found it hard to wind down”), which are scored on a four-point scale ranging from 0 = “Did not apply to me at all” to 3 = “Applied to me very much or most of the time” in relation to the past week. The depression subscale evaluates symptoms such as hopelessness, dysphoria, lack of interest, and self-deprecation. The anxiety subscale evaluates situational anxiety and the subjective experience of anxious affect. The stress subscale estimates the level of chronic non-specific arousal. Lower scores indicate a lower level of psychological distress. In the present study, the internal consistency (Cronbach’s alpha) for this scale was 0.91.

**The Brief Resilience Scale (BRS)** was developed by Smith et al. [43, 44] to assess the ability to bounce back or recover from stress. The Azerbaijani version of the BRS was conducted by Rustamov et al. [45]. BRS is a self-report scale consisting of six items (“It is hard for me to snap back when something bad happens”). Items are rated on a 5-point Likert-type (from 1 = “Strongly disagree” to 5 = “Strongly agree”) measurement tool. Cronbach’s alpha of the scale was found 0.87.

### Translation

The translation procedure was carefully aligned with standardized protocols, following the guidelines outlined by Beaton et al. [46]. Initially, two bilingual translators independently translated the original English version of the WHO-5 Well-being Index into Azerbaijani. After this step, a detailed comparison of the two translations was conducted to identify and resolve any differences, through discussion and agreement among the translators and the research team. A committee, consisting of the translators and research team members, then reviewed the translated version to make necessary adjustments, ensuring cultural appropriateness and clarity. The revised Azerbaijani version was back-translated into English by another bilingual translator, who was not informed of the original version to ensure objectivity. This back-translated version was compared to the original to identify and address any discrepancies. The final Azerbaijani version of the WHO-5 Well-being Index was developed and later subjected to psychometric evaluation.

### Data analysis

Confirmatory factor analysis (CFA) employing maximum likelihood estimation was conducted using AMOS Graphics 24 for both the WHO-5 Well-being Index. Model fit was evaluated using Comparative Fit Index (CFI), Normed Fit Index (NFI), Incremental Fit Index (IFI), and Standardized Root Mean Square Residual (SRMR). Furthermore, the item-total correlations of the scale were examined. To assess convergent validity, the Average Variance Extracted (AVE) was computed.

To enhance the validation process, we employed Item Response Theory (IRT) to model the WHO-5 Well-being Index, utilizing the Graded Response Model (GRM) within Stata 15. Additionally, we computed various reliability coefficients, including Cronbach’s alpha (α), McDonald’s omega (ω), and Guttmann’s lambda (λ6). In addition, composite reliability (CR) was calculated.

Furthermore, we examined the association between WHO-5 Well-being Index and depression, anxiety, stress, psychological resilience, and life satisfaction. The relationships were assessed using correlation coefficients. In addition, we conducted a comprehensive network analysis that encompassed all these variables, with the aim of visually representing the interconnections among them. This network analysis was carried out using JASP 0.18.1 to provide a holistic view of the associations among the variables under investigation. Descriptive statistics, correlations, and assumption tests were also conducted using IBM SPSS Statistics 22.

## Results

**Fig. 1:**
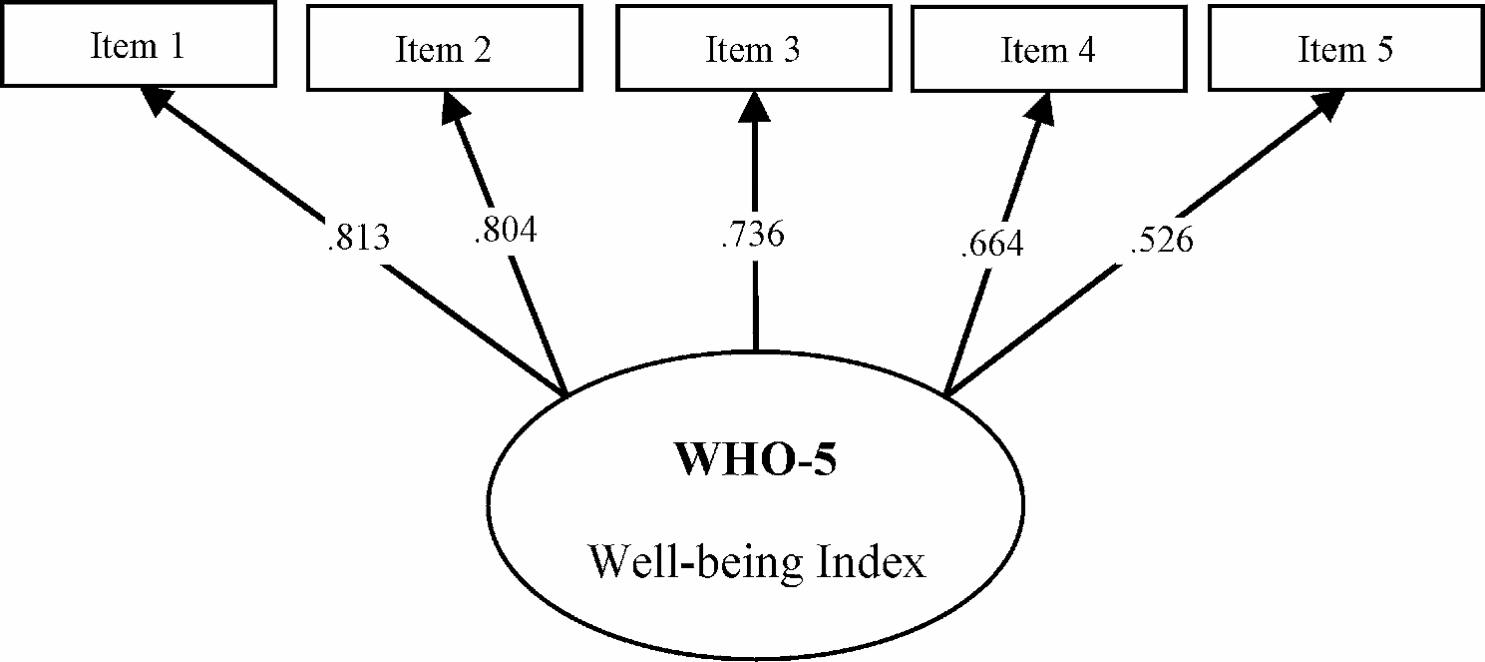
Structure validity of the Azerbaijani WHO-5 well-being index

For the assessment of normality in the dataset’s distribution, skewness and kurtosis were analyzed. Skewness was found to be 0.275, and kurtosis was − 0.733, positioning both metrics comfortably within the accepted ranges for normal distribution in social sciences, as established by Kline [47] (-2 to + 2 for skewness) and West et al. [48] (-3 to + 3 for kurtosis). Through this analysis, a symmetric distribution of the variables was confirmed, indicating their compliance with the criteria of normality. The results of the Confirmatory Factor Analysis (CFA) for the WHO-5 Well-being Index indicated a favorable model fit: χ² (5, *N* = 875) = 53.797; Goodness of Fit Index (GFI) = 0.975; Adjusted Goodness of Fit Index (AGFI) = 0.925; Relative Fit Index (RFI) = 0.935; Incremental Fit Index (IFI) = 0.971; Comparative Fit Index (CFI) = 0.970; Standardized Root Mean Square Residual (SRMR) = 0.031. The unidimensional factor model of the 5-item scale accounted for 51.320% of the total variance, with standardized factor loadings ranging from 0.526 to 0.813 (see Fig. 1). Convergent validity was confirmed through standardized loadings and the use of Average Variance Extracted (AVE), which stood at 0.513, exceeding the 0.50 benchmark set by Bagozzi and Yi [49]. The statistical analysis and parameter estimates further affirm the study’s latent constructs’ convergent validity.

Following the confirmation of the scale’s structure, Item Response Theory (IRT) analysis was conducted. Table 2 presents the results, showing that the discrimination parameter (α) values ranged from 1.224 to 2.998. Consistent with Baker’s [50] guidelines, 4 items were classified as having a very high level of discrimination, while one item remained classified as moderate. These findings underscore the high discriminative power of the WHO-5 Well-being Index, indicating its efficacy in distinguishing between varying levels of wellbeing.

**Table 2:** IRT results for the WHO-5 well-being index

Item	*a* coefficient	SE	Confidence interval	*z*	*p* >|*z*|
I have felt cheerful in good spirits. *Özümü yaxşı* ə*hval-ruhiyy*ə*d*ə*, ş*ə*n hiss etmiş*ə*m.*	2.998	0.211	2.585–3.412	14.21	0.001
I have felt calm and relaxed. *Özümü sakit v*ə *rahatlamış hiss etmiş*ə*m.*	2.785	0.187	2.417–3.153	14.84	0.001
I have felt active and vigorous. *Özümü aktiv v*ə *enerjik hiss etmiş*ə*m.*	2.164	0.137	1.894–2.433	15.75	0.001
I woke up feeling fresh and rested. *S*ə*h*ə*rl*ə*r özümü gümrah v*ə *dinc*ə*lmiş hiss ed*ə*r*ə*k oyanmışam.*	1.791	0.118	1.559–2.024	15.12	0.001
My daily life has been filled with things that interest me. *Günd*ə*lik h*ə*yatım m*ə*ni maraqlandıran şeyl*ə*rl*ə *doludur.*	1.224	0.090	1.047–1.400	13.59	0.001

The internal consistency reliability of the scale was rigorously evaluated using three distinct coefficients: Cronbach’s alpha, McDonald’s omega, and Guttmann’s lambda. The results consistently demonstrated robust reliability. In particular, Cronbach’s alpha coefficient yielded a value of 0.829, highlighting the scale’s commendable reliability. Furthermore, the McDonald’s omega coefficient, another reliable measure, yielded a value of 0.829. Additionally, the Guttmann’s lambda coefficient produced a value of 0.814, confirming that the items within the scale effectively measure the same underlying construct. The composite reliability value (CR), indicative of the constructs’ measurement accuracy through their items, was found to be 0.838, surpassing the 0.70 threshold for acceptability. This demonstrates that the latent variables within the study possess dependable measurement attributes.

In terms of criterion-related validity, the analysis revealed several significant correlations with WHO-5 Well-being Index (see Table 3). WHO-5 Well-being Index exhibited negative correlations with depression (*r* = −.485, *p* <.001), anxiety (*r* = −.336, *p* <.001), and stress (*r* = −.422, *p* <.001). Furthermore, there were positive associations between WHO-5 Well-being Index and both psychological resilience (*r* =.396, *p* <.001) and life satisfaction (*r* =.565, *p* <.001).

**Table 3:** Relationship of the WHO-5 wellbeing index with the variables

Variable	Correlation with WHO-5 Wellbeing Index		95% Confidence Interval	
	*r*	*p*	*LL*	*UL*
Depression	− 0.485	< 0.001	− 0.526	− 0.441
Anxiety	− 0.336	< 0.001	− 0.394	− 0.276
Stress	− 0.422	< 0.001	− 0.475	− 0.366
Psychological resilience	0.396	< 0.001	0.339	0.450
Life satisfaction	0.565	< 0.001	0.503	0.621

**Fig. 2:**
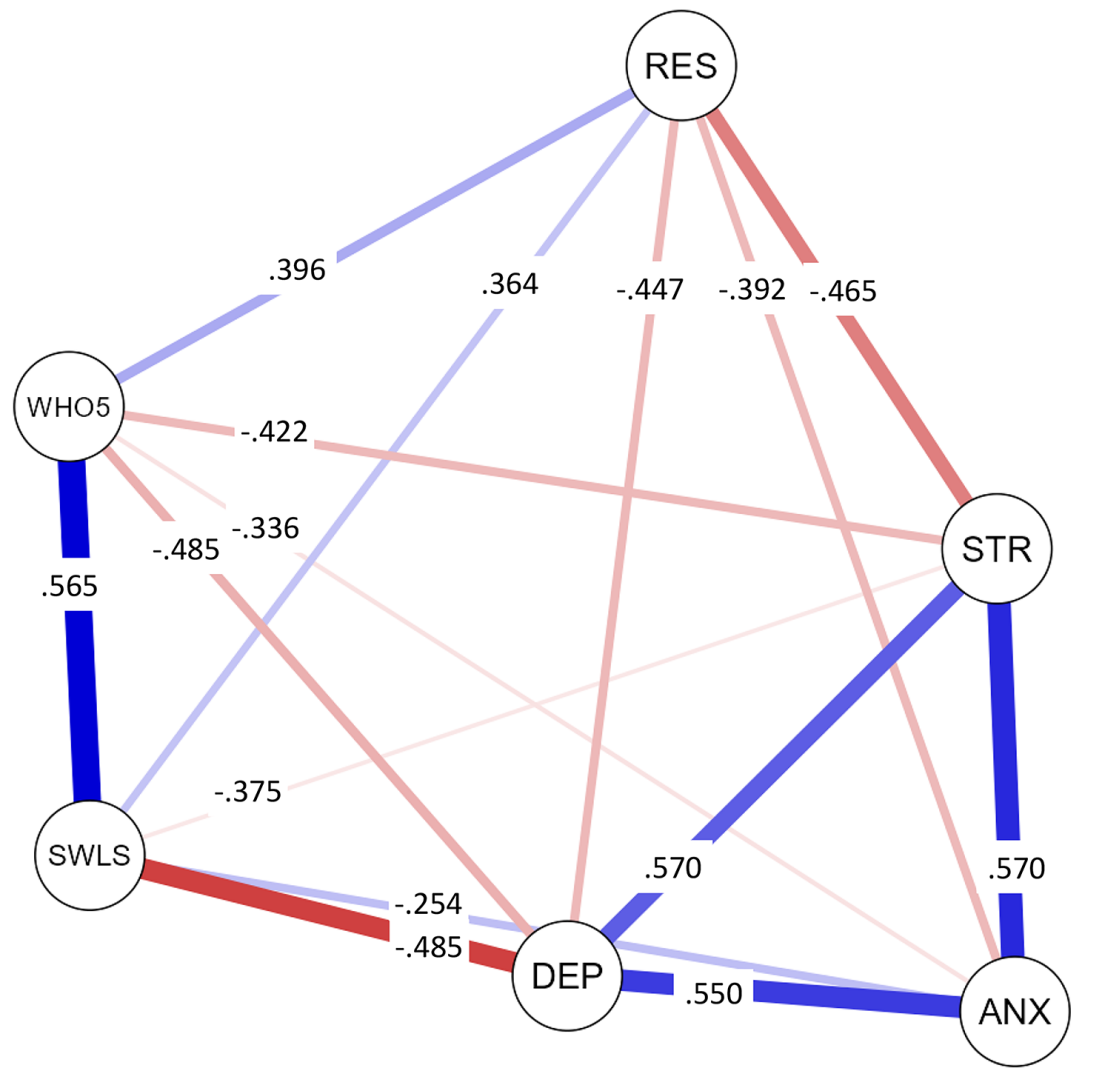
Network analysis results

The results of the network analysis, depicted in Fig. 2, illustrate the relationships between WHO-5 Well-being Index and other variables. Notably, WHO-5 Well-being Index exhibited strong connections with life satisfaction, depression, and psychological resilience, underscoring the significant associations between these constructs.

## Discussion

Researching individuals’ well-being is crucial for identifying factors that influence life quality, life satisfaction, and for mitigating adverse consequences for people, thereby enhancing the level of psychological health. Hence, the WHO-5 Well-being Index Scale is essential to explore factors affecting personal, academic, and career life, as well as psychological health. The WHO-5 Well-being Index Scale is the most widely utilized tool for measuring well-being in many countries around the globe. It has been translated into 30 languages (including Chinese, Polish, Thai, etc.) and adapted to various cultures [30–33], demonstrating its significance in assessing subjective well-being levels. In this study, we examined the psychometric properties, such as validity and reliability, of the WHO-5 Well-being Index Scale within the Azerbaijani culture. The primary aim of this research was to adapt the scale to the Azerbaijani language, to assess its validity and reliability, and to explore the relationship between the well-being index and life satisfaction, subjective well-being, depression, anxiety, stress, and psychological resilience.

The psychometric findings from the confirmatory factor analysis (CFA) of the Azerbaijani-adapted version of the scale affirmed the structure of the original version. The analysis demonstrated that the 5-item self-rated scale enables individuals to assess their level of well-being based on symptoms experienced over a 14-day period. These findings align with those from other adaptations [31, 32] of the WHO-5 Well-being Index, particularly concerning the scale’s 5 items. The results indicate that the scale possesses satisfactory psychometric properties in terms of internal consistency and reliability.

The WHO-5 Well-being Index underwent psychometric evaluation using various methods and samples. Analyzes aimed at assessing the scale’s internal consistency revealed that its reliability level exceeded 0.70 for the total score, aligning with Nunnally and Bernstein’s benchmark, which considers a Cronbach’s alpha above 0.70 as sufficient [51]. For this scale, Cronbach’s alpha was precisely 0.829. Beyond Cronbach’s alpha, additional reliability assessments were conducted using McDonald’s omega and Gutmann’s Lambda. In this study, McDonald’s omega was calculated to be 0.829, Gutmann’s lambda was 0.814, and the composite reliability score reached 0.838.

Despite its validation and reliability in various languages, the WHO-5 Well-being Index’s applicability in the Azerbaijani language, particularly its association with life satisfaction, depression, anxiety, distress, and resilience, was explored. Considering the anticipated outcomes, the presence of a well-validated scale in Azerbaijani facilitates culturally relevant assessments, leading to more accurate and meaningful results. This is essential for a deeper understanding of individuals’ psychological states and for identifying potential concerns like depression, anxiety, and stress. Moreover, adapting this scale enables comprehensive research on well-being within the Azerbaijani context. It was observed that individuals with higher levels of well-being are less prone to negative emotions such as anxiety, stress, and sadness, indicating a negative correlation between well-being and depression, anxiety, and stress [28, 52, 53]. Furthermore, results showed that increased life satisfaction and positive emotions are associated with higher well-being indexes. Additionally, the findings suggest a positive correlation between individuals’ psychological resilience, subjective well-being, and the well-being index [10, 52].

Overall, this study provided valuable insights into the psychometric properties, reliability, and validity of the WHO-5 Well-being Index Scale within the Azerbaijani context. The results obtained are consistent with prior research, affirming the scale’s reliability and criterion-related validity. This emphasizes the significant influence of well-being on the mental health of the Azerbaijani population.

### Future directions

The validation of the WHO-5 Well-being Index for the Azerbaijani population offers a robust foundation for future research directions. Key areas of focus include exploring the relationship between well-being and various factors such as individual, social, and occupational elements. Identifying groups with high well-being and those at risk will enable targeted interventions and support mechanisms. Developing and testing interventions for at-risk groups using the WHO-5 Well-being Index is crucial. This approach will assess the interventions’ effectiveness, contributing to evidence-based practices tailored to the Azerbaijani context. Longitudinal research is essential to understand the trajectory of well-being over time. Investigating the reasons behind changes in well-being can offer insights into the impact of societal changes, policy interventions, and personal life events. Additionally, the adaptation of the WHO-5 Well-being Index enables participation in cross-cultural studies, enhancing our understanding of well-being in a global context. This will allow for the comparison of well-being factors across different cultures and the development of universal strategies to improve psychological health.

### Limitations

The present study has typical limitations that are required to be taken into consideration when interpreting the results. The first limitation of the study is that findings were skewed toward females which raises concerns about generalizability of the findings, as well-being index and mental health can differ in terms of gender. Second, the majority of research participants had attained a higher education level, which may limit the generalizability of the findings regarding well-being and life satisfaction to populations with varying educational backgrounds. Third, the data were cross-sectional which only consented for associations to be observed and causality between variables could not be established. Fourth, the absence of test-retest reliability assessment in our study limits our ability to confirm the scale’s stability over time, marking a significant limitation. Lastly, participants selected randomly without any clinical characteristics, therefore generalizing the results to the broader population may not be acceptable.

## Conclusion

In conclusion, this study offers detailed insights into the psychometric properties and validity of the WHO-5 Well-being Index within the Azerbaijani context, particularly through its associations with life satisfaction, depression, anxiety, stress, psychological resilience, and subjective well-being. The results affirm that the scale is a reliable tool for assessing the well-being index in Azerbaijan, notwithstanding its limitations. The findings from this study are poised to assist psychologists, healthcare professionals, and policymakers in conducting further research, thereby facilitating a deeper understanding of well-being and its consequential effects on adult mental health.

## Data Availability

No datasets were generated or analysed during the current study.

## References

[1] Rustamov E, Zalova Nuriyeva U, Allahverdiyeva M, Abbasov T, Mammadzada G, Rustamova N (2023). Academic self-efficacy, academic procrastination, and well-being: a mediation model with a large sample of Azerbaijan. IOJPE.

[2] Diener E (2009). Assessing well-being: the collected works of Ed Diener, 331.

[3] Huppert FA (2009). Psychological well-being: evidence regarding its causes and consequences. Appl Psychol Health Well-Being.

[4] Myers D, Diener E (1995). Who is happy? Psychol. Sci.

[5] Ronen T, Hamama L, Rosenbaum M, Mishely-Yarlap A (2016). Subjective well-being in adolescence: the role of Self-Control, Social Support, Age, gender, and Familial Crisis. J Happiness Stud.

[6] Diener E, Suh EM, Lucas RE, Smith HL (1994). Subjective well-being: three decades of progress. Psychol Bull.

[7] Maker-Castro E, Wray-Lake L, Cohen AK (2022). Critical consciousness and wellbeing in adolescents and young adults: a systematic review. Adolesc Res Rev.

[8] Huppert FA, So TTC (2013). Flourishing across Europe: application of a new conceptual framework for defining well-being. Soc Indic Res.

[9] Ruggeri K, Garcia-Garzon E, Maguire Á, Matz S, Huppert FA (2020). Well-being is more than happiness and life satisfaction: a multidimensional analysis of 21 countries. Health Qual Life Outcomes.

[10] Diener E (2012). New findings and future directions for subjective well-being research. Am Psychol.

[11] Oishi S, Diener E, Lucas RE (2007). The optimum level of well-being: can people be too happy?. Perspect Psychol Sci.

[12] Richards M, Huppert FA (2011). Do positive children become positive adults? Evidence from a longitudinal birth cohort study. J Posit Psychol.

[13] Aliyev BH (2020). COVID-19 pandemia influence on human behavior. J Psychol.

[14] UNICEF. Adolescent health and well-being. 2020 https://www.unicef.org/health/adolescent-health-and-well-being (accessed June 19th 2023).

[15] Deniz ME, Arslan U, Satici B, Kaya Y, Akbaba MF (2023). A Turkish adaptation of the fears and resistances to Mindfulness Scale: factor structure and psychometric properties. J Social Educational Res.

[16] Scheier MF, Carver CS (1992). Effects of optimism on psychological and physical well-being: theoretical overview and empirical update. Cognit Ther Res.

[17] Boehm JK, Kubzansky LD (2012). The heart’s content: the association between positive psychological well-being and cardiovascular health. Psychol Bull.

[18] Carver CS (1998). Resilience and thriving: issues, models, and linkages. J Soc Issues.

[19] Jackson D, Firtko A, Edenborough M (2007). Personal resilience as a strategy for surviving and thriving in the face of workplace adversity: a literature review. J Adv Nurs.

[20] Kutuk H (2023). Investigating the role of resilience as a mediator in the link between internet addiction and anxiety. J Social Educational Res.

[21] Wright MO, Masten AS, Narayan AJ, Goldstein S, Brooks RB (2013). Resilience processes in development: four waves of research on positive adaptation in the context of adversity. Handbook of Resilience in Children.

[22] Connor KM, Davidson JR (2003). Development of a new resilience scale: the Connor-Davidson resilience scale (CD-RISC). Depress Anxiety.

[23] Ong AD, Bergeman CS, Bisconti TL, Wallace KA (2006). Psychological resilience, positive emotions, and successful adaptation to stress in later life. J Pers Soc Psychol.

[24] Mak Winnie WS, Ivy NG, WONG Celia SW (2011). Resilience: enhancing well-being through the positive cognitive triad. J Couns Psychol.

[25] De Wit M, Pouwer F, Gemke RJ, Delemarre-van de Waal HA, Snoek FJ (2003). Validation of the WHO-5 well-being index in adolescents with type 1 diabetes. Diabetes Care.

[26] Quansah F, Hagan JE, Francis A, Agormedah EK, Nugba RM, Srem-Sai M, Schack T (2022). Validation of the WHO-5 well-being scale among adolescents in Ghana: evidence-based Assessment of the Internal and External structure of the measure. Children.

[27] Kusier AO, Folker AP (2019). The Well-Being Index WHO-5: hedonistic foundation and practical limitations. Med Humanit.

[28] Topp CW, Østergaard SD, Søndergaard S, Bech P (2015). The WHO-5 well-being index: a systematic review of the literature. Psychother Psychosom.

[29] Newnham EA, Hooke GR, Page AC (2010). Monitoring treatment response and outcomes using the World Health Organization’s Wellbeing Index in psychiatric care. J Affect Disord.

[30] Perera BPR, Jayasuriya R, Caldera A, Wickremasinghe AR (2020). Assessing mental well-being in a Sinhala speaking Sri Lankan population: validation of the WHO-5 well-being index. Health Qual Life Outcomes.

[31] Fung S-f, Kong CYW, Liu Y-m, Huang Q, Xiong Z, Jiang Z (2022). Validity and psychometric evaluation of the Chinese Version of the 5-Item WHO well-being index. Front Public Health.

[32] Omani-Samani R, Maroufizadeh S, Almasi-Hashiani A, Sepidarkish M (2019). The WHO-5 well-being index: a validation study in people with infertility. Iran J Public Health.

[33] Nylén-Eriksen M, Bjørnnes AK, Hafstad H, Lie I, Grov EK, Lara-Cabrera ML (2022). Validating the five-Item World Health Organization Well-Being Index. IJERPH.

[34] Saipanish R, Lotrakul M, Sumrithe S (2009). Reliability and validity of the Thai version of the WHO-Five Well-Being Index in primary care patients. PCN.

[35] Lara-Cabrera ML, Betancort M, Muñoz-Rubilar A, Rodríguez-Novo N, Bjerkeset O (2022). De Las Cuevas C. Psychometric properties of the WHO-5 well-being index among nurses during the COVID-19 pandemic: a cross-sectional study in three countries. IJERPH.

[36] World Health Organization (WHO.) Use of well-being measures in primary health care-the DepCare project health for all. Stockholm, Sweden: 1998.

[37] Diener E, Emmons RA, Larsen RJ, Griffin S (1985). The satisfaction with Life Scale. J Pers Assess.

[38] Rustamov E, Musayeva T, Xalilova X, Ismayilova G, Nahmatova U (2023). Investigating the links between social support, psychological distress, and life satisfaction: a mediation analysis among Azerbaijani adults. J Posit Psychol Wellbeing.

[39] Osmanli N, Babayev A, Rustamov I, Munir KM (2021). Psychometric evaluation of the satisfaction with Life Scale (SWLS) in Azerbaijan. J Educ Train Stud.

[40] Lovibond SH, Lovibond PF. Depression anxiety stress scales (DASS–21, DASS–42) [Database record]. APA Psych Tests; 1995.

[41] Rustamov E, Nahmatova U, Aliyeva M, Mammadzada G, Asadov F (2023). The impact of emotional intelligence on teachers’ job satisfaction: mediating role of psychological distress. Int J Educ Sci.

[42] Rustamov E, Zalova Nuriyeva U, Allahverdiyeva M, Abbasov T, Rustamova N (2023). Azerbaijani Adaptation of the Perceived School Experience Scale: examining its impact on psychological distress and school satisfaction. Probl Educ 21st Century.

[43] Smith BW, Dalen J, Wiggins K, Tooley E, Christopher P, Bernard J (2008). The brief resilience scale: assessing the ability to bounce back. Int J Behav Med.

[44] Rustamov E, Aliyeva M, Nahmatova U, Asadov F, Mammadzada G (2023). Relations among psychological resilience, exam anxiety, and school satisfaction in a large sample of Azerbaijani adolescents. Eur J Educ Res.

[45] Rustamov E, Musayeva T, Xalilova X, Ismayilova G, Nahmatova U (2023). Exploring the relationship between social connectedness and mental wellbeing: the mediating role of psychological resilience among adults in Azerbaijan. Discov Psychol.

[46] Beaton DE, Bombardier C, Guillemin F, Ferraz MB (2000). Guidelines for the process of cross-cultural adaptation of self-report measures. Spine.

[47] Kline RB. Principles and practice of structural equation modeling. Guilford; 2023.

[48] West SG, Finch JF, Curran PJ. Structural equation models with nonnormal variables: problems and remedies. In: Hoyle RH, editor. Structural equation modeling: concepts, issues, and applications. Sage Publications, Inc; 1995. pp. 56–75.

[49] Bagozzi RP, Yi Y. The degree of intention formation as a moderator of the attitude-behavior relationship. Soc Psychol Q. 1989; 266–79.

[50] Baker C (2001). Foundations of Bilingual Education and Bilingualism.

[51] Nunnally JC, Bernstein IH (1994). The Assessment of Reliability. Psychometric Theory.

[52] Klainin-Yobas P, Vongsirimas N, Ramirez DQ, Sarmiento J, Fernandez Z (2021). Evaluating the relationships among stress, resilience and psychological well-being among young adults: a structural equation modelling approach. BMC Nurs.

[53] Grant CA, Wallac LM, Spurgeon PC (2013). An exploration of the psychological factors affecting remote e-worker’s job effectiveness, well-being and work-life balance. Empl.

